# GATA3 interacts with and stabilizes HIF-1α to enhance cancer cell invasiveness

**DOI:** 10.1038/onc.2017.8

**Published:** 2017-03-06

**Authors:** M-C Lin, J-J Lin, C-L Hsu, H-F Juan, P-J Lou, M-C Huang

**Affiliations:** 1 Department of Otolaryngology, National Taiwan University Hospital, Hsin-Chu Branch, Hsinchu City, Taiwan; 2 Graduate Institute of Anatomy and Cell Biology, National Taiwan University College of Medicine, Taipei, Taiwan; 3 Institute of Biochemistry and Molecular Biology, National Taiwan University College of Medicine, Taipei, Taiwan; 4 Department of Life Science, National Taiwan University, Taipei, Taiwan; 5 Department of Otolaryngology, National Taiwan University Hospital and College of Medicine, Taipei, Taiwan

## Abstract

GATA binding protein 3 (GATA3) is indispensable in development of human organs. However, the role of GATA3 in cancers remains elusive. Hypoxia inducible factor (HIF)-1 plays an important role in pathogenesis of human cancers. Regulation of HIF-1α degradation is orchestrated through collaboration of its interacting proteins. In this study, we discover that GATA3 is upregulated in head and neck squamous cell carcinoma (HNSCC) and is an independent predictor for poor disease-free survival. GATA3 promotes invasive behaviours of HNSCC and melanoma cells *in vitro* and in immunodeficient mice. Mechanistically, GATA3 physically associates with HIF-1α under hypoxia to inhibit ubiquitination and proteasomal degradation of HIF-1α, which is independent of HIF-1α prolyl hydroxylation. Chromatin immunoprecipitation assays show that the GATA3/HIF-1α complex binds to and regulates HIF-1 target genes, which is also supported by the microarray analysis. Notably, the GATA3-mediated invasiveness can be significantly reversed by HIF-1α knockdown, suggesting a critical role of HIF-1α in the underlying mechanism of GATA3-mediated effects. Our findings suggest that GATA3 stabilizes HIF-1α to enhance cancer invasiveness under hypoxia and support the GATA3/HIF-1α axis as a potential therapeutic target for cancer treatment.

## Introduction

GATA3, a zinc-finger transcription factor belonging to the GATA family,^1, 2^ is first identified to be critical in T-cell differentiation.^2, 3^ Later on, it is discovered to have pivotal roles in development of various human organs.^4, 5, 6, 7, 8, 9^ The association of GATA3 with human malignancies has been reported in breast cancers, neuroblastomas, endometrial carcinomas, urothelial carcinomas and soft tissue sarcomas.^10, 11, 12, 13, 14^ However, its function remains elusive.

The hypoxia inducible factor-1 (HIF-1) is a heterodimer composed of α and β subunits. Whereas HIF-1β is constitutively expressed, HIF-1α undergoes rapid degradation in the presence of oxygen.^15^ The oxygen labile nature of HIF-1α is regulated by a family of prolyl hydroxylases (PHDs), which target two proline residues (Pro 402 and Pro 564) of HIF-1α in an oxygen-dependent manner.^16^ Hydroxylated HIF-1α allows the recognition of the von Hippel-Lindau (VHL)-E3 ligase complex, leading to proteasomal degradation of HIF-1α.^17^ Besides the PHD-VHL-dependent degradation, compelling evidence supports that other mediators regulate HIF-1α degradation.^18^ Tumour hypoxia and increased HIF-1α expression are associated with disease progression, chemoradioresistance and increased patient mortality in certain cancers.^19, 20^ Therefore, understanding the mechanism of HIF-1α regulation is key to unveiling tumour pathophysiology and to providing new strategies for cancer therapy.^21^

Here, we identify the physical interaction between GATA3 and HIF-1α and demonstrate that GATA3 inhibits the proteasomal degradation of HIF-1α under hypoxia. In clinical samples, GATA3 expression positively correlates with HIF-1α and is an independent predictor for poor disease-free survival in head and neck squamous cell carcinoma (HNSCC). Gene expression profiling shows a significant overlap between GATA3 and HIF-1α regulated genes in HNSCC cells. These data strongly indicate that GATA3 regulates the HIF-1α-mediated cellular response to promote tumour progression under hypoxia.

## Results

### GATA3 is overexpressed in HNSCC and predicts poor prognosis

**Figure 1:**
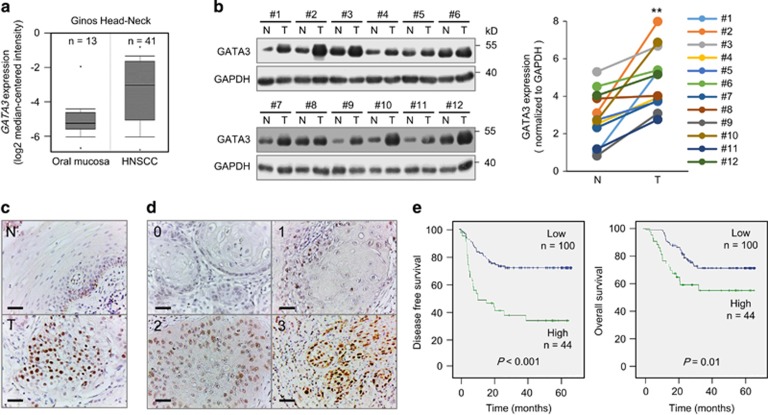
GATA3 is overexpressed in HNSCC and high GATA3 expression is associated with poor survivals. (**a**) *GATA3* mRNA is overexpressed in HNSCC. *GATA3* mRNA levels are higher in HNSCC (*n*=41) compared with normal oral mucosa (*n*=13). The data are retrieved from Ginos Head-Neck in the Oncomine database (https://www.oncomine.org). (**b**) Western blot analysis of GATA3 expression in paired HNSCC tumours (T) and adjacent non-tumour mucosa tissues (N) from 12 patients. Left: Western blots showing GATA3 expression and GAPDH control. Right: GATA3 expression was quantified and analysed by paired Student’s *t*-test. **P<0.01. (**c**) Representative images of immunohistochemistry of GATA3 in paired adjacent non-tumour mucosa (N) and tumour tissues (T) from HNSCC patients (*n*=15). Scale bars, 50 μm. (**d**) Scoring of immunohistochemistry of GATA3 in HNSCC tumour tissues. The intensity of GATA3 staining is scored from 0 to 3 and grouped into low (score=0, 1) and high (score=2, 3) expression. (**e**) Kaplan–Meier survival analysis. Patients with follow-up period over 18 months are included (*n*=144). Left and right panels indicate disease-free and overall survivals of patients with low and high GATA3 expression, respectively.

From Oncomine database, we noticed higher GATA3 expression in HNSCC than that in normal oral mucosa (Figure 1a). Western blotting of 12 paired non-tumour/tumour tissues from HNSCC patients confirmed the GATA3 overexpression in HNSCC tumours (Figure 1b, *P*<0.01). Immunohistochemical staining also showed that GATA3 was overexpressed in HNSCC tumours compared with adjacent non-tumour epithelia (Figure 1c). We thus extended our study to evaluate GATA3 expression in 151 HNSCC tumours. In these tumours, the expression levels of GATA3 were categorized into low (score 0 and 1) and high (score 2 and 3; Figure 1d). Results showed that GATA3 was highly expressed in 31% (47/151) of the HNSCC tumours (Table 1). High GATA3 expression significantly correlated with adverse prognostic factors including lymph node metastasis, distant metastasis, high grade, perineural invasion, lymphovascular invasion, extracapsular spread and presence of locoregional recurrence. Cox regression analysis showed that GATA3 is an independent predictor for poor disease-free survival (Supplementary Table S1, *P*=0.02). Kaplan–Meier analysis showed that patients with higher GATA3 expression had poorer disease-free and overall survivals (Figure 1e, *P*<0.001 and *P*=0.01, respectively). These results suggest that GATA3 is overexpressed in HNSCC tumours and is associated with poor clinical outcomes.

### GATA3 promotes tumour invasiveness *in vitro* and *in vivo*

**Figure 2:**
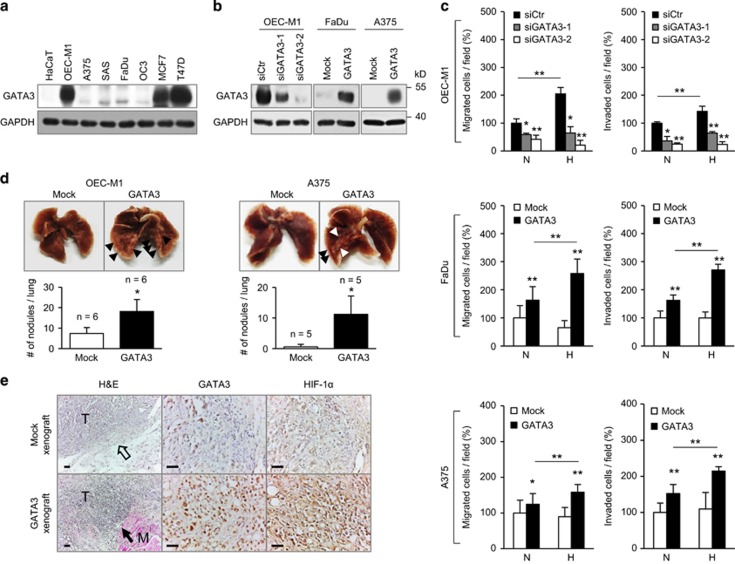
GATA3 promotes tumour invasiveness *in vitro* and *in vivo*. (**a**) Western blot analysis of GATA3 expression in various cell lines as indicated. GAPDH was an internal control. (**b**) Knockdown and overexpression of GATA3 were confirmed by western blot analysis. For knockdown, OEC-M1 cells were transfected with non-targeting siRNA (siCtr) or two independent siRNAs against *GATA3* (siGATA3-1 and siGATA3-2). For overexpression, FaDu and A375 cells were transfected with empty pcDNA3.1 (Mock) or *GATA3*/pcDNA3.1 (GATA3). (**c**) Effects of GATA3 on cell migration and invasion analysed by transwell migration and Matrigel invasion assays, respectively. Cells were seeded after transfection for 48 h. Normoxia (N, O_2_=21%) or hypoxia (H, O_2_=1%) is as indicated. Results are represented as mean±s.d. from three independent experiments. *P<0.05, **P<0.01. (**d**) Effects of GATA3 on experimental metastasis *in vivo*. Upper panel, representative images of lungs and metastatic tumour nodules indicated by arrowheads. Lower panel, statistical analysis of tumour nodules. Mock or GATA3 overexpressing OEC-M1 and A375 stable transfectants (1 × 10^6^ cells / per injection) were injected through tail veins into NOD-SCID mice (*n*=6 and 5, respectively). Results are represented as mean±s.d. Data are analysed by Student’s *t*-test. *P<0.05. (**e**) Expression of GATA3 and HIF-1α in subcutaneous tumour xenografts. Left, hematoxylin and eosin staining. The hollow arrow indicates the intact capsule separating the tumour from surrounding tissues. The black arrow indicates the invasion of tumour into skeletal muscles. M, skeletal muscle. T, tumour. Middle and right, immunohistochemical staining of GATA3 and HIF-1α. Scale bars, 50 μm.

Because results from clinical samples showed that GATA3 expression was associated with tumour invasiveness, we next investigated the effects of GATA3 on cell migration and invasion by transwell migration and Matrigel invasion assays, respectively. Western blotting showed varied expression levels of GATA3 in keratinocytes (HaCaT), HNSCC cells (OEC-M1, SAS, FaDu and OC3), melanoma cells (A375) and breast cancer cells (MCF7 and T47D) (Figure 2a). OEC-M1 cells were chosen for GATA3 knockdown (Figure 2b). In addition, FaDu and A375 cells were chosen for GATA3 overexpression. Results showed that GATA3 knockdown significantly decreased migration and invasion in OEC-M1 cells, while GATA3 overexpression significantly increased migration and invasion in FaDu and A375 cells (Figure 2c). Because hypoxia is a strong inducer for tumour invasiveness, particularly in HNSCC and melanoma,^22, 23, 24^ we next performed migration and invasion assays under 1% O_2_. Interestingly, GATA3 knockdown abolished the hypoxia-induced migration and invasion in OEC-M1 cells, while GATA3 overexpression enhanced the migration and invasion to a greater extent in both FaDu and A375 cells under hypoxia (Figure 2c). MTT assays showed that GATA3 did not significantly affect cell viability during the test period for migration and invasion assays (Supplementary Figure S1a). These results suggest that GATA3 enhances cancer cell invasiveness and these effects are more pronounced under hypoxia.

Next, we investigated the effects of GATA3 on tumour invasiveness and growth *in vivo* in immunodeficient mice. We established stable clones of GATA3 overexpressing OEC-M1 and A375 cells as well as GATA3 knockdown OEC-M1 cells (Supplementary Figure S1b). Results of experimental metastasis models showed that GATA3 overexpression in OEC-M1 and A375 cells significantly increased whereas GATA3 knockdown in OEC-M1 cells significantly decreased lung colonization of tumour cells (Figure 2d and Supplementary Figure S1c). Moreover, results of tumour xenograft models showed that GATA3 overexpression in OEC-M1 cells significantly enhanced tumour growth *in vivo* (Supplementary Figure S1d). It is worth noting that GATA3 overexpressing tumours invaded into adjacent skeletal muscles, whereas control tumours were entirely enveloped by capsules (Figure 2e, left). Echoing with *in vitro* finding that GATA3 augmented cell invasiveness under hypoxia, immunohistochemistry of tumour xenografts showed that expression of HIF-1α was significantly increased in GATA3 overexpressing tumours compared with control tumours (Figure 2e, middle and right and Supplementary Figure S1e). Moreover, tumour angiogenesis, one of the most recognized HIF-1-mediated response,^25^ was significantly increased in GATA3 overexpressing xenografts as shown by CD31 staining (Supplementary Figure S1f). These results suggest that GATA3 promotes tumour invasiveness *in vitro* and *in vivo* and HIF-1α may contribute to the underlying mechanism.

To evaluate whether there is correlation between GATA3 and tumour cell proliferation in clinical samples, we performed IHC of Ki-67 in tumours from patients operated from year 2013 to 2014 (*n*=56). Representative images of Ki-67 score are shown in Supplementary Figure S2a. The results showed no significant correlation between GATA3 and Ki-67 expression (Supplementary Figure S2b). Moreover, we also performed MTT assay to assess the effect of GATA3 on cell viability over 72 h. The results showed that knockdown of GATA3 significantly suppressed the viability of OEC-M1 cells (Supplementary Figure S2c). However, overexpression of GATA3 slightly suppressed the viability of FaDu cells. The effect of GATA3 on the viability of A375 cells was not significant. These results indicate that GATA3 does not significantly affect cell proliferation in HNSCC tumours, and its effects on cell viability are cell-context dependent.

### GATA3 stabilizes HIF-1α proteins

**Figure 3:**
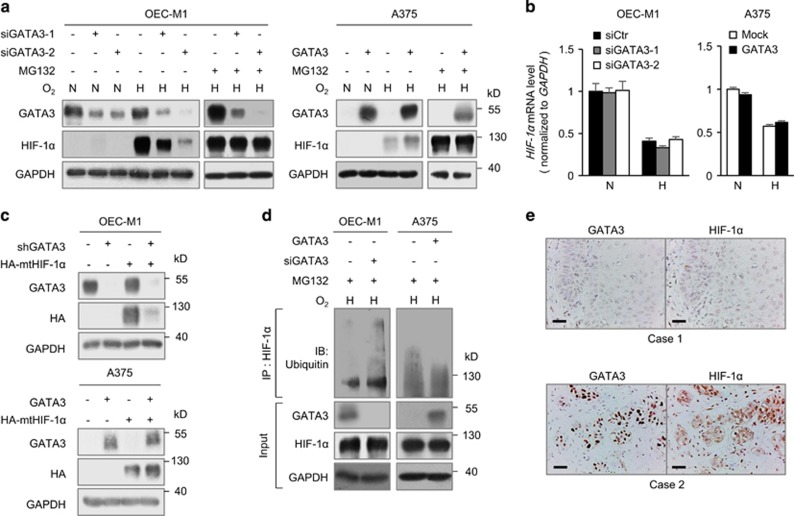
GATA3 expression increases HIF-1α protein levels. (**a**) Western blot analysis of GATA3 and HIF-1α under normoxia (N) or hypoxia (H) for overnight with or without a proteasome inhibitor, MG132 (20 μM), as indicated. GAPDH was an internal control. OEC-M1 cells were transfected with non-targeting siRNA or *GATA3* siRNA (siGATA3-1 or siGATA3-2) and A375 cells were transfected with Mock or GATA3 plasmid. (**b**) Real-time RT-PCR analysis of *HIF-1α* mRNA expression. The total RNA was extracted from cells grown under normoxia or hypoxia for overnight. The results are represented as mean±s.d. from three independent experiments. (**c**) Western blot analysis of GATA3 and P402A/P564A HIF-1α mutant. GAPDH was an internal control. pLKO or sh*GATA3*/pLKO (shGATA3) expressing OEC-M1 stable transfectants as well as Mock or GATA3 overexpressing A375 stable transfectants were transfected with empty vector or HA-P402A/P564A *HIF-1α*/pcDNA3.1 (HA-mtHIF-1α). (**d**) Effects of GATA3 on HIF-1α ubiquitination. OEC-M1 cells transfected with non-targeting siRNA or *GATA3* siRNA (siGATA3-2) and A375 cells transfected with Mock or GATA3 plasmid were incubated with MG132 (20 μM) under hypoxia for 6 h. Cell lysates were immunoprecipitated with anti-HIF-1α antibody and then immunoblotted with anti-ubiquitin antibody. GATA3, HIF-1α and GAPDH in whole cell lysates (input) were shown. (**e**) Representative images of immunohistochemical staining of GATA3 and HIF-1α in serial sections of HNSCC tissues. Scale bars, 50 μm.

We next investigated the effect of GATA3 on HIF-1α expression by western blotting. Under hypoxia or with CoCl_2_, GATA3 knockdown decreased HIF-1α expression in OEC-M1 and T47D cells (Figure 3a, left panel and Supplementary Figure S3a and b). In contrast, HIF-1α was higher in A375, FaDu and OEC-M1 cells overexpressing GATA3 compared with their controls (Figure 3a, right panel and Supplementary Figure S3a and b). We also found that MG132, a proteasome inhibitor, blocked the influence of GATA3 on HIF-1α protein levels (Figure 3a). Real-time RT-PCR analysis showed that *HIF-1α* mRNA was not significantly changed by GATA3 (Figure 3b). These results suggest that GATA3 increases HIF-1α protein levels by inhibiting its proteasomal degradation.

To clarify whether the effect of GATA3 on HIF-1α degradation is PHD-dependent, P402A/P564A HIF-1α mutant was transiently expressed in OEC-M1, A375, T47D and 293FT cells. The results showed that GATA3 knockdown decreased whereas GATA3 overexpression increased levels of P402A/P564A HIF-1α mutant (Figure 3c and Supplementary Figure S3c), indicating that the regulation of GATA3 on HIF-1α degradation is PHD-independent. Furthermore, protein degradation assays showed that the half-life of HIF-1α protein was significantly shorter in GATA3 knockdown OEC-M1 cells compared with control cells (47.8 vs 108.8 min) (Supplementary Figure S3d). Immunoprecipitation assays showed that, under hypoxia with MG132, GATA3 overexpression in A375 cells decreased whereas GATA3 knockdown in OEC-M1 cells increased the levels of ubiquitinated HIF-1α (Figure 3d).

Next, we asked whether GATA3 and HIF-1α were co-expressed in cells and clinical samples. Consistent with the western blotting data, confocal immunofluorescence microscopy showed GATA3 knockdown decreased whereas GATA3 overexpression increased the fluorescence intensity of HIF-1α in cells (Supplementary Figure S4a and b). We further investigated the correlation between GATA3 and HIF-1α expression in the 151 HNSCC tumours by immunohistochemistry, and the results confirmed a significantly positive correlation (Pearson’s correlation coefficient=0.54, *P*<0.001; Supplementary Figure S5a). The representative images were shown in Figure 3e. Among the 151 tumours, 49 tumours were positive for both GATA3 and HIF-1α staining. In these GATA3 and HIF-1α double-positive tumours, we further categorized the distribution of positive staining into two types: invasive front and diffuse. Chi-square analysis showed a significant correlation between GATA3 and HIF-1α distribution (Supplementary Table S2, *P*<0.01). Representative images of IHC for GATA3 and HIF-1α from serial sections are shown in Supplementary Figure S5b. Western blotting of 12 HNSCC tumours consistently showed a significantly positive correlation of GATA3 with HIF-1α and GATA3 with SLC2A1, a well-known HIF-1 target (Pearson’s correlation coefficient=0.65 and 0.64, respectively, *P*<0.05; Supplementary Figure S5c). Moreover, high HIF-1α expression significantly correlates with decreased disease-free and overall survivals in 144 HNSCC patients (Supplementary Figure S5d). Collectively, these results suggest that GATA3 stabilizes HIF-1α protein by inhibiting the proteasomal degradation pathway and GATA3 expression positively correlates with HIF-1α expression in clinical samples.

### GATA3 physically interacts with HIF-1α

**Figure 4:**
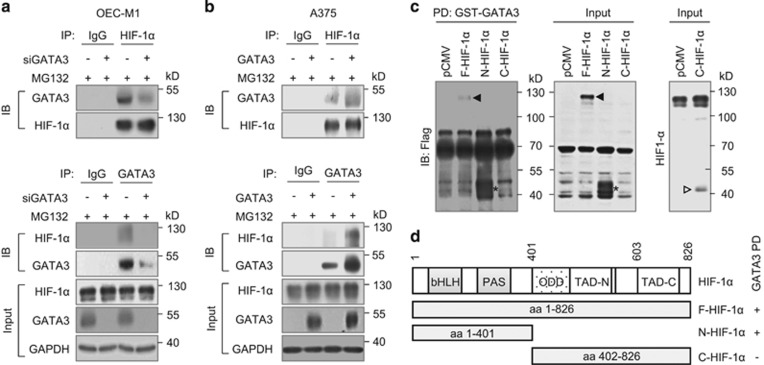
GATA3 physically interacts with HIF-1α. (**a**, **b**) Co-immunoprecipitation assays of GATA3 and HIF-1α in OEC-M1 and A375 cells. OEC-M1 cells were transfected with non-targeting siRNA or *GATA3* siRNA (siGATA3-2) and A375 cells were transfected with Mock or GATA3 plasmid. Cells were incubated with MG132 (20 μM) and CoCl_2_ (800 μM) for 6 h. Upper panel, cell lysates were immunoprecipitated with IgG control antibody or anti-HIF-1α antibody and then immunoblotted with anti-GATA3 or anti-HIF-1α antibody. Lower panel, cell lysates were immunoprecipitated with IgG control antibody or anti-GATA3 antibody and then immunoblotted with anti-HIF-1α or anti-GATA3 antibody. HIF-1α, GATA3 and GAPDH in whole cell lysates (input) were shown. (**c**) GST pull-down assays. 293FT cells were transfected with *F-*, *N-* or *C-HIF-1α*/pCMV-Tag 4A (Flag-tagged) and incubated with CoCl_2_ (800 μM) for 4 h. Left panel, GST-GATA3 fusion protein pulled down F-HIF-1α and N-HIF-1α, but not C-HIF-1α. On the same membrane, C-HIF-1α could not be detected by the rabbit anti-HIF-1α polyclonal antibody (Cell Signaling Technology), which is known to recognize this region (Ser653, data not shown). The black arrowhead indicates F-HIF-1α. The asterisk indicates N-HIF-1α. The expression of F-HIF-1α and N-HIF-1α was confirmed by western blotting and shown in the middle panel. For unknown reason, C-HIF-1α could not be detected by anti-Flag antibody. We used rabbit anti-HIF-1α polyclonal antibody (Cell Signaling Technology) to confirm C-HIF-1α expression as shown in the right panel (hollow arrowhead). (**d**) The design of HIF-1α constructs is based on known functional domains and the pull-down assay results were summarized schematically. PAS, Per-ARNT-Sim.

To understand the mechanism of GATA3-mediated HIF-1α stabilization, we next assessed whether GATA3 could physically interact with HIF-1α. Co-immunoprecipitation assays showed that GATA3 and HIF-1α existed in the same protein complex in OEC-M1 and A375 cells (Figures 4a and b). More convincingly, GATA3 knockdown decreased whereas GATA3 overexpression increased the association between GATA3 and HIF-1α. To further specify the binding domain of HIF-1α with GATA3, we generated truncated mutants of HIF-1α and performed GST pull-down assays. Results showed that GATA3 interacted with the full-length and the N-terminal part of HIF-1α (N-HIF-1α, aa 1-401), which contains the basic helix-loop-helix and Per-ARNT-Sim domains (Figures 4c and d). In contrast, the C-terminal part (C-HIF-1α, aa 402–826) did not interact with GATA3. Taken together, these results suggest that GATA3 physically interacts with HIF-1α.

### GATA3 regulates the expression of HIF-1 target genes

**Figure 5:**
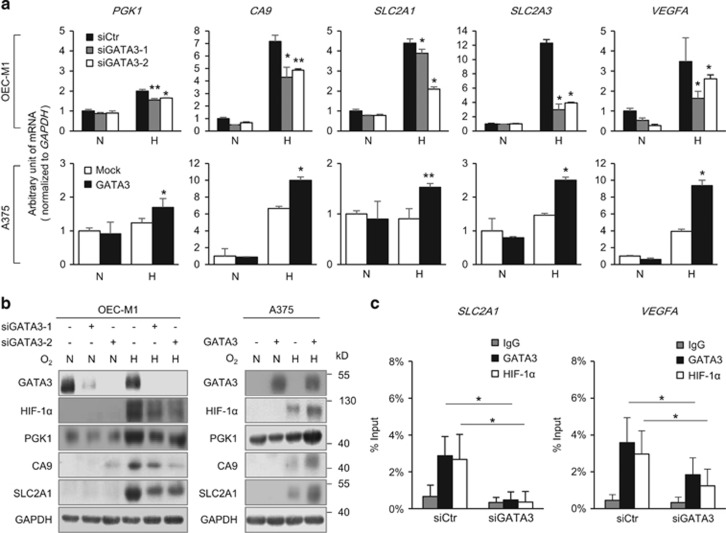
GATA3 regulates expression of HIF-1 target genes under hypoxic conditions. (**a**) Real-time RT-PCR analysis of the expression of HIF-1α target genes, including *PGK1*, *CA9*, *SLC2A1, SLC2A3* and *VEGFA* in OEC-M1 and A375 cells. (**b**) Western blot analysis of GATA3, HIF-1α, PGK1, CA9 and SLC2A1. GAPDH was an internal control. Total RNAs and cell lysates were extracted from cells grown under normoxia (N) or hypoxia (H), as indicated. (**c**) ChIP assays with anti-GATA3 or anti-HIF-1α antibody and *SLC2A1* and *VEGFA* genes in OEC-M1 cells. Control or GATA3 knockdown cells were cultured under hypoxia for overnight and then harvested for ChIP assays. Isotype matched IgG was used as controls. The results are represented as mean±s.d. from three independent experiments. *P<0.05, **P<0.01.

Because GATA3 stabilizes HIF-1α, we next analysed whether GATA3 could regulate the expression of well-known HIF-1 target genes, including *PGK1*, *CA9*, *SLC2A1*, *SLC2A3* and *VEGFA.*^26^ Real-time RT-PCR analysis showed that, under hypoxia, the transcripts of these genes were significantly decreased in GATA3 knockdown OEC-M1 cells and increased in GATA3 overexpressing A375 cells (Figure 5a). In contrast, no significant difference in their mRNA expression was observed under normoxia. Western blot analysis confirmed that GATA3 knockdown decreased whereas GATA3 overexpression increased PGK1, CA9 and SLC2A1 under hypoxia (Figure 5b). To elucidate how GATA3 regulated HIF-1 target genes, chromatin immunoprecipitation (ChIP) assays were performed in OEC-M1 cells. Results showed that both GATA3 and HIF-1α were associated with the hypoxia response element on *SLC2A1* and *VEGFA* promoters (Figure 5c). Moreover, GATA3 knockdown significantly decreased the association of GATA3 and HIF-1α with these promoters. These results suggest that GATA3 regulates the expression of HIF-1 target genes by physically interacting with HIF-1α.

### GATA3-mediated cancer cell invasiveness is reversed by HIF-1α knockdown or N-HIF-1α expression

**Figure 6:**
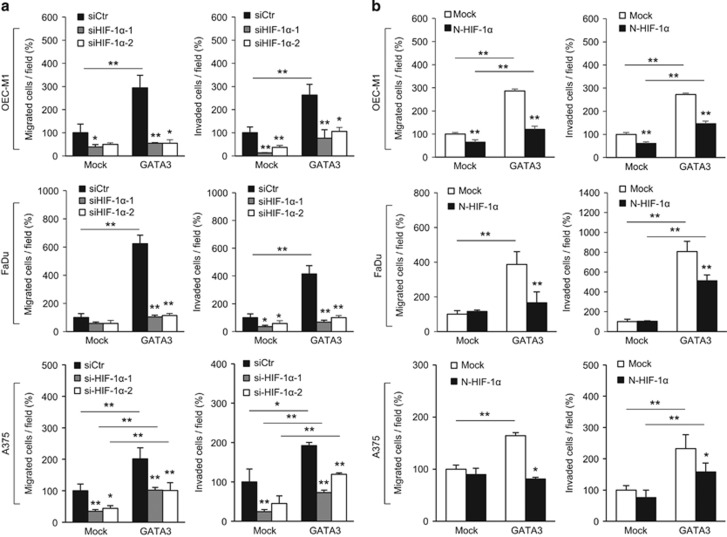
GATA3-mediated cancer cell invasiveness is reversed by HIF-1α knockdown or N-HIF-1α expression. (**a**) Effects of HIF-1α knockdown on migration and invasion in OEC-M1, FaDu and A375 cells overexpressing GATA3. Mock or GATA3 overexpressing OEC-M1, FaDu and A375 stable transfectants were transiently transfected with non-targeting siRNA (siCtr) or two independent siRNAs against *HIF-1α* (siHIF-1α-1, siHIF-1α-2). Transwell migration and Matrigel invasion assays were carried out under hypoxia. (**b**) Effects of the ectopic N-HIF-1α expression on GATA3-mediated migration and invasion. Mock or GATA3 overexpressing OEC-M1, FaDu and A375 stable transfectants were transfected with empty vector (Mock) or *N-HIF-1α*/pCMV-Tag 4A (N-HIF-1α). Transwell migration and Matrigel invasion assays were carried out under hypoxia. The results are represented as mean±s.d. from three independent experiments. *P<0.05, **P<0.01.

To assess the contribution of HIF-1α to the GATA3-mediated cancer cell invasiveness, we knocked down endogenous HIF-1α or expressed N-HIF-1α in Mock or GATA3 stable transfectants and then carried out transwell migration and Matrigel invasion assays under hypoxia. Results showed that silencing *HIF-1α* with siRNA significantly decreased the GATA3-induced migration and invasion of OEC-M1, FaDu and A375 cells (Figure 6a). Given that GATA3 can bind to N-HIF-1α, we tested whether this construct without transactivation domain could block GATA3-mediated cancer cell invasiveness. Results showed that ectopic expression of N-HIF-1α significantly reversed GATA3-induced migration and invasion under hypoxia (Figure 6b). The expression of HIF-1α or N-HIF-1α was shown in Supplementary Figure S6. These results suggest that HIF-1α contributes to the GATA3-mediated cancer cell invasiveness under hypoxia and that blocking the GATA3/HIF-1α interaction is sufficient to suppress the invasiveness.

### The enriched functions of GATA3/HIF-1α axis are critical for tumour malignancy

To unravel the functional pathways regulated by the GATA3/HIF-1α axis in response to hypoxia, we performed cDNA microarray analysis in GATA3 or HIF-1α knockdown OEC-M1 cells. The results showed that 1408 and 2714 genes were differentially expressed in GATA3 knockdown and HIF-1α knockdown cells, respectively (Supplementary Figure S7a). Of which, 287 genes were significantly overlapped (*P*=3.4e-21). The gene ontology enrichment analysis indicated that these overlapping genes are mostly responsible for cell proliferation, migration and regulation of secretion, which are critical for tumour malignancy and progression (Supplementary Figure S7b). The differential expression of selected genes, including *TGFB2, IGFBP3, ARHGDIB, ERBB3, LEF1, HMG20B* and *WASF3*, was validated by real-time RT-PCR analysis (Supplementary Figure S8). The individual functional maps of GATA3 and HIF-1α were shown in Supplementary Figure S9. Although the differentially expressed genes may represent both direct and indirect targets of GATA3 and HIF-1α, these results support that the GATA3/HIF-1α axis plays an important role in regulating tumour malignancy under hypoxia.

## Discussion

Here, we report that GATA3 functions as an HIF-1α regulator to promote cancer cell invasiveness. Our data show that GATA3 expression is upregulated in HNSCC and high GATA3 expression correlates with lymph node metastasis, distant metastasis, high grade, presence of perineural invasion, lymphovascular invasion, nodular extracapsular spreads and locoregional recurrence. In accordance, GATA3 promotes cancer cell invasiveness *in vitro* and *in vivo*. GATA3-mediated invasiveness is enhanced under hypoxia and significantly reversed by HIF-1α knockdown or ectopic expression of N-HIF-1α (aa 1-401). Mechanistic investigation indicates that GATA3 physically interacts with and inhibits proteasomal degradation of HIF-1α. The stabilized HIF-1 complex then regulates its target genes to promote malignant behaviours of cancer cells. A significant overlap between GATA3 and HIF-1α-regulated genes by microarray analysis further supports a critical role of HIF-1α in GATA3-mediated effects under hypoxia.

The role of GATA3 in tumour malignancy varies in different cancers. GATA3 expression is associated with favourable disease-free and overall survivals in breast cancers.^10^ However, GATA3 is a poor prognostic marker in neuroblastomas, endometrial carcinomas and soft tissue sarcomas.^11, 12, 14^ The underlying mechanisms of the distinct function of GATA3 still remain unclear. It has been reported that, in breast cancer cells, GATA3 forms a complex with G9A/NuRD (MTA3) to suppress *ZEB2*, *TGFB1* and other epithelial-to-mesenchymal transition-related genes.^27^ By contrast, in luminal-type breast cancer cells, GATA3 promotes cell cycles by physically interacting with poly-ADP ribose polymerase 1 (PARP1) and subsequently activating transcription of *CCND1.*^28^ Interestingly, GATA3 overexpression decreases mammosphere numbers in human mammary epithelial cells but increases the numbers in MCF-7 cells, suggesting that GATA3 effect shifts from tumour suppressing to tumour promoting during tumorigenesis.^29^ In the present study, we discover that GATA3 associates with and stabilizes HIF-1α, rendering transcriptional changes of HIF-1 target genes and an increased tumour invasiveness under hypoxia. These findings implicate that GATA3 has disparate functions in distinct cancer types, depending on the differential interaction with specific cofactors under genetic/epigenetic context *per se*.

Although the regulation of GATA3 on HIF-1α responsive genes is critical for tumour invasiveness under hypoxia and is emphasized in this study, GATA3 also regulates non-HIF-1α responsive genes, as shown in our functional pathway analysis. These genes, which could be transcriptional targets of GATA3, may work cooperatively with those in the HIF-1 pathway, and partly contribute to the GATA3-mediated tumour malignancy under hypoxia. In addition, it is worth mentioning that our results also show that GATA3 enhances cancer cell invasiveness under normoxia. Therefore, to understand the thorough function of GATA3 during the entire process of tumour progression, it will be of great interest to identify the transcriptional targets of GATA3 under normoxia.

GATA3 increases levels of HIF-1α under hypoxia or in the presence of hypoxic-mimetic agent, CoCl_2_. In addition, GATA3 stabilizes P402A/P564A HIF-1α mutant, which is resistant to hydroxylation by PHD. These results indicate that GATA3 stabilizes HIF-1α independently of PHD. Several PHD-independent regulators of HIF-1α have been reported, including HAF, MDM2, Hsp90 and RACK1,^30, 31, 32^ many of which bind to the N-terminal half of HIF-1α. Here, we report that GATA3 physically interacts with aa 1-401 of HIF-1α. It is possible that GATA3 competes with one or more PHD-independent regulators for binding to HIF-1α and therefore protects HIF-1α from proteasomal degradation. Moreover, we found that, in clinical samples of HNSCC, the expression and distribution of GATA3 and HIF-1α are significantly correlated, implying that GATA3 can interact with HIF-1α in tumour cells to regulate their behaviours *in vivo*. Although GATA3 expression is not sufficient to overcome oxygen-dependent HIF-1α degradation under normoxia, we demonstrate that GATA3 is critical in stabilizing HIF-1α under hypoxia.

In accordance with previous studies,^23, 33^ we also found that high HIF-1α expression significantly correlates with decreased disease-free and overall survivals in HNSCC patients. As accumulating evidence supports that HIF-1 is instrumental for tumour adaption to hypoxic microenvironment,^21, 34^ our findings bring up innovation in the aspect of cancer therapy. These results suggest that targeting GATA3 or blocking the GATA3/HIF-1α interaction could be a novel strategy to inhibit the HIF-1 pathway. Recently, targeting GATA3 is clinically applicable owing to the emergence of *GATA3* DNA enzyme (DNAzyme).^35, 36^ In addition, small molecule compounds or peptides designed to block the interaction of GATA3 with HIF-1α would be logical and promising to counteract the GATA3/HIF-1α axis.

In conclusion, this is the first study to thoroughly describe the expression and clinical significance of GATA3 in HNSCC. We discover that, in addition to functioning as a transcription factor, GATA3 interacts with and stabilizes HIF-1α protein under hypoxia. Our findings add on current understanding of the delicate yet complex regulation of HIF-1α degradation and shed light on the pursuit of prognostic markers and therapeutic targets of HNSCC.

## Materials and methods

### Tissue samples

Human tissues from HNSCC patients operated from year 2010 to 2014 were obtained from National Taiwan University Hospital, Taipei, Taiwan. The use of human tissues was approved by the local hospital ethics committee, and written consents were obtained from all patients. (IRB numbers: 201304078RIND, 201307074RIND).

### Immunohistochemistry

Paraffin embedded tissues were incubated with anti-human GATA3 monoclonal antibody (1:100, Santa Cruz Biotechnology, Dallas, TX, USA), anti-human HIF-1α monoclonal antibody (1:200, Novus Biologicals, Cambridge, MA, USA), or anti-mouse CD31 polyclonal antibody (1:50, LifeSpan BioSciences, Seattle, WA, USA) at 4 °C for overnight. The UltraVision Quanto Detection System (Thermo Fisher Scientific, Waltham, MA, USA) was used and signals were visualized with DAB Quanto Chromogen provided in the same kit. All sections were counterstained with hematoxylin. For assessment of intensity, each field was graded semi-quantitatively on tree-tier scale where 0=none staining, 1=weak staining, 2=moderate staining, 3=strong staining. Scoring was evaluated by investigators who were blinded to the clinical information.

### Plasmid construction

The human full-length *GATA3* (NM_002051) cDNA clone was purchased from OriGene (SC108486, Rockville, MD, USA) and subcloned into pcDNA3.1/mycHis plasmid using the restriction sites of EcoRI and XbaI. The full-length, N-terminal (encoding aa 1-401) and C-terminal (encoding aa 402-826) human HIF-1α (NM_001530) were obtained from OEC-M1 cells by RT-PCR. The PCR products were cloned into pCMV-Tag 4A. HA-P402A/P564A *HIF-1α*/pcDNA3.1 was obtained from Addgene (Cambridge, MA, USA) (Dr William Kaelin’s laboratory, plasmid #18955).^37^ sh*GATA3*/pLKO was obtained from National RNAi Core Facility (Academia Sinica, Taipei, Taiwan, TRCN0000273991). All the constructs were verified by DNA sequencing.

### Cell cultures and transfection

HaCaT, SAS, A375, OEC-M1 and OC3 cells were gifts from Dr Jean-San Chia (National Taiwan University). FaDu cells were purchased from Bioresource Collection and Research Center (Hsinchu, Taiwan). T47D cells were gifts from Dr Tang-Long Shen (National Taiwan University Hospital). 293FT cells were purchased from Thermo Fisher Scientific. All cell lines were authenticated by STR DNA profiling analysis in the year 2016. Cells were transiently transfected with 2.5 μg of plasmid with Lipofetamine 3000 (Invitrogen, Carlsbad, CA, USA) according to the manufacturer’s protocol. Stable clones were selected with G418 (400 μg/ml) or puromycin (1 μg/ml) for 14 days. For GATA3 and HIF-1α knockdown, cells were transfected with 20 nM of siRNAs against *GATA3* (siGATA3-1: 5′-CCACACUCUGGAGGAGGAAUGCCAA-3′, siGATA3-2: 5′-CCCGCCUCUGCUUCAUGGAUCCCUA-3′), *HIF-1α* (siHIF-1α-1: 5′-CCAGCCGCUGGAGACACAAUCAUAU-3′, or siHIF-1α-2: 5′-GGGAUUAACUCAGUUUGAACUAACU-3′) using Lipofectamine RNAiMAX (Invitrogen). Non-targeting siRNA (si-Control, 5′-CAACCUCAGCCAUGUCGACUGGUUU-3′, 5′-AAACCAGUCGACAUGGCUGAGGUUG-3′) was used as control.

### Transwell migration assay and Matrigel invasion assay

Transwell migration assay was performed by using migration chambers with an 8-μm pore size membrane (Corning, Corning, NY, USA). Matrigel invasion assay was performed according to the BioCoat Matrigel Invasion Chamber system (BD Biosciences, San Jose, CA, USA). Cells (2 × 10^4^) were re-suspended in serum-free DMEM and added to an upper chamber. Lower chambers were loaded with 10% fetal bovine serum and cells were allowed to migrate or invade for 24 h under 1 or 21% O_2_. Migrated or invaded cells on the lower surface of the membrane were fixed with 100% methanol and then stained with 5% crystal violet (Sigma-Aldrich, St Louis, MO, USA). The number of migrated or invaded cells per field was counted under a phase contrast microscope. The mean±s.d. was calculated from the number of six different fields.

### Animals

All animal experiments were approved by Institutional Animal Care and Use Committee of National Taiwan University College of Medicine, Taipei, Taiwan. Female NOD-SCID mice (8 weeks) were obtained from the National Laboratory Animal Center, Taipei, Taiwan.

### RNA extraction and real-time RT-PCR

Total RNA was extracted with GeneJET RNA purification kit (Thermo Fisher Scientific) according to the manufacturer’s protocol. Two micrograms of total RNA were used in reverse transcription reaction using the High-Capacity cDNA Reverse Transcription Kit (Applied Biosystems, Carlsbad, CA, USA). The cDNA was subjected to real-time RT-PCR. Primer sequences were listed in Supplementary Table S3. Relative quantity of gene expression was normalized to *GAPDH* and analysed with MxPro Software (Stratagene, La Jolla, CA, USA).

### Western blot analysis and co-immunoprecipitation

Tissue and whole cell lysates were extracted by homogenizing tissues or cell pellets in RIPA buffer. For co-immunoprecipitation, lysates containing 500 μg of total protein were incubated with specific antibodies (2 μg) for 18 hours at 4 °C with constant rotation. After that, 50 μl of protein G agarose beads were added to incubate for 3 h. The beads were washed five times with lysis buffer. The precipitated proteins were resuspended in 30 μl SDS sample buffer and boiled at 95 °C for 10 min. Proteins were separated on an 8% SDS-PAGE and transferred onto PVDF membrane. The membranes were blocked in 5% bovine serum albumin (BSA, Bio-Rad, Hercules, CA, USA) for 1 h at room temperature and incubated with primary antibodies against GATA3, HIF-1α, CA9, SLC2A1, HA-tag (Cell Signaling Technology, Danvers, MA, USA), HAF (Thermo Fisher Scientific), PGK1, GAPDH (Santa Cruz Biotechnology) or Flag-tag (Sigma-Aldrich) at 4 °C for overnight. The membranes were then incubated with horseradish peroxidase conjugated secondary antibodies and the protein bands were detected by ECL reagents (GE Healthcare Life Sciences, Pittsburgh, PA, USA).

### GST pull-down assay

To obtain GST-GATA3 fusion protein, human full-length *GATA3* was subcloned into pGEX-4T-3 (Amersham, Piscataway, NJ, USA) and the construct was expressed in BL21DE3 *E. coli.* The expression of soluble GST-GATA3 was confirmed by mass spectrometry. *E. coli* lysates containing GST-GATA3 were incubated with Glutathione-Sepharose 4 Fast Flow beads (GE Healthcare Life Sciences) at 4 °C for 2 h to generate the GST-GATA3 conjugated beads. Lysates of cells expressing different constructs of HIF-1α were incubated with GST-GATA3 conjugated beads at 4 °C for 18 h and then washed with lysis buffer for five times. The pulled down proteins were eluted by SDS sample buffer and analysed by western blotting.

### Chromatin immunoprecipitation assay (ChIP)

After incubation under 1% O_2_ for 24 h, OEC-M1 cells were harvested and the ChIP experiments were performed according to the manufacturer’s protocol (Thermo Fisher Scientific). The primers used were listed in Supplementary Table S3. Real-time RT-PCR analysis was performed by using the SYBR Green master mix (Thermo Fisher Scientific).

### Statistics

Statistical analysis was performed by using two-sided Student’s *t*-test. Chi-square test was used to analyse the correlation between GATA3 expression and clinicopathological characteristics. Kaplan–Meier and Cox regression analysis were performed for survival analyses. Pearson’s correlation test was used to assess the correlation between two proteins expression in primary HNSCC tissues. *P*<0.05 is considered statistically significant.

## Figures and Tables

**Table 1:** Chi-square test of GATA3 scores with patient characteristics

*Characteristics*	*GATA3 score*		P *value*
	*Low*	*High*	
Age ⩽ 60	67	29	0.75
Age>60	37	18	
			
Male	99	44	0.69
Female	5	3	
			
Oral cavity	73	29	0.25
Oropharynx	10	2	
Hypopharynx	15	11	
Larynx	6	5	
			
T1–2	57	20	0.16
T3–4	47	27	
			
N0–1	85	27	0.002*
N2–3	19	20	
			
M0	96	34	0.001*
M1	8	13	
			
Low grade	56	11	< 0.001*
High grade^a^	48	36	
			
LVI −	84	29	0.01*
LVI +	20	18	
			
PNI −	69	22	0.02*
PNI +	35	25	
			
ECS −	22	6	0.004*
ECS+^b^	13	18	
			
LR −	81	24	0.001*
LR+^c^	19	20	

Abbreviations: ECS, extracapsular spread; LR, locoregional recurrence; LVI, lymphovascular invasion; PNI, perineural invasion.

*P<0.05.

a Low grade: grade 1, high grade: grade 2–3.

b Only patients with positive nodal metastasis (*n*=59) were selected for ECS analysis.

c Only patients followed for more than 18 months (*n*=144) were included in analysis for LR.

## Supplementary Material

Supplementary Table S2

Supplementary Table S3 and Materials and Method
