# Erratum: GATA3 interacts with and stabilizes HIF-1α to enhance cancer cell invasiveness

**DOI:** 10.1038/onc.2017.196

**Published:** 2017-06-12

**Authors:** M-C Lin, J-J Lin, C-L Hsu, H-F Juan, P-J Lou, M-C Huang

**Keywords:** Transcription factors, Oncogenesis, Head and neck cancer, Disease-free survival

**Correction to:** *Oncogene* (2017) **36,** 4243–4252; doi: 10.1038/onc.2017.8; published online 6 March 2017

The authors noted an error in the bottom panel of Figure 2c. A revised version is shown below and the text of the manuscript remains unchanged.

**Figure 1:**
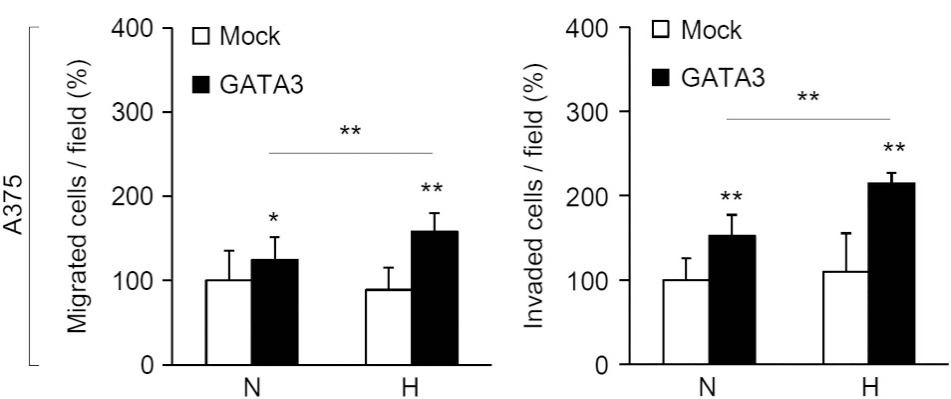
Figure 2

The authors would like to apologize for any inconvenience caused and confirm that the conclusions drawn from this research are not affected by this error. 

