# Supplementary material for: GATA3 interacts with and stabilizes HIF-1α to enhance cancer cell invasiveness
Source: Oncogene. 2017 Mar 6;36(30):4243–52. doi: 10.1038/onc.2017.8 (PMC5537608; doi:10.1038/onc.2017.8)
Supplement: Supplementary Figure S1-S9 [file onc20178x1.pdf]

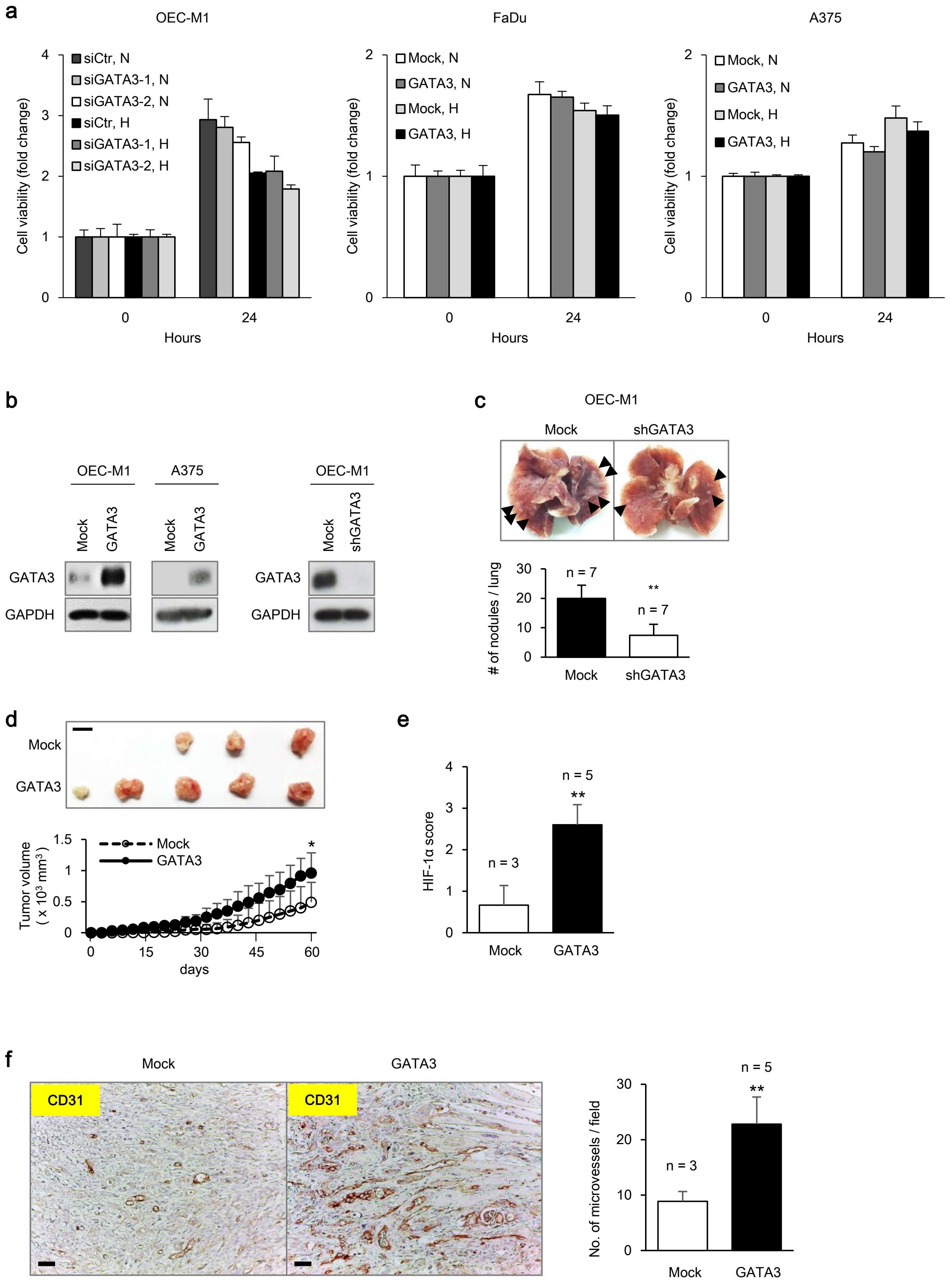

Supplementary Figure S1

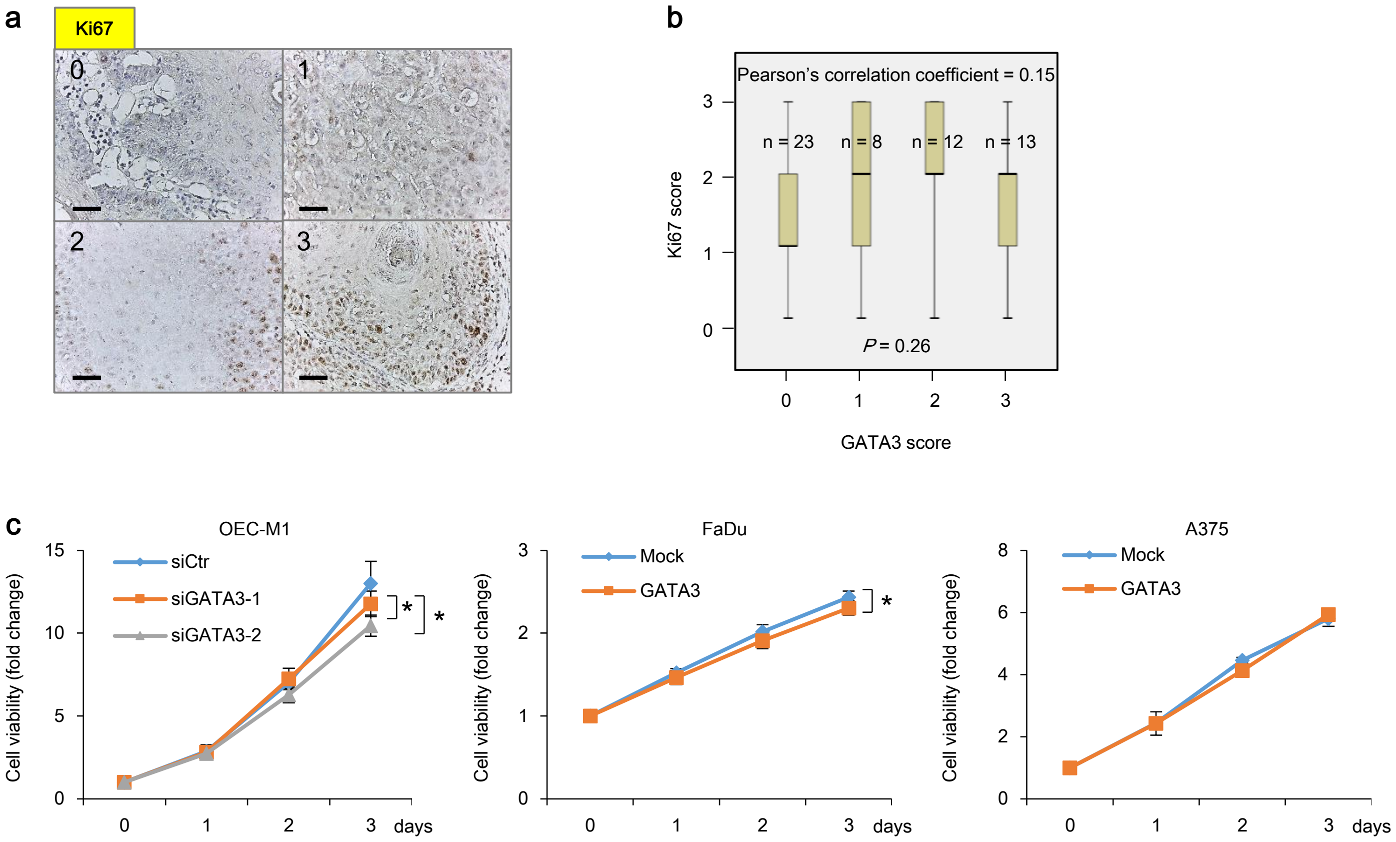

Supplementary Figure S2

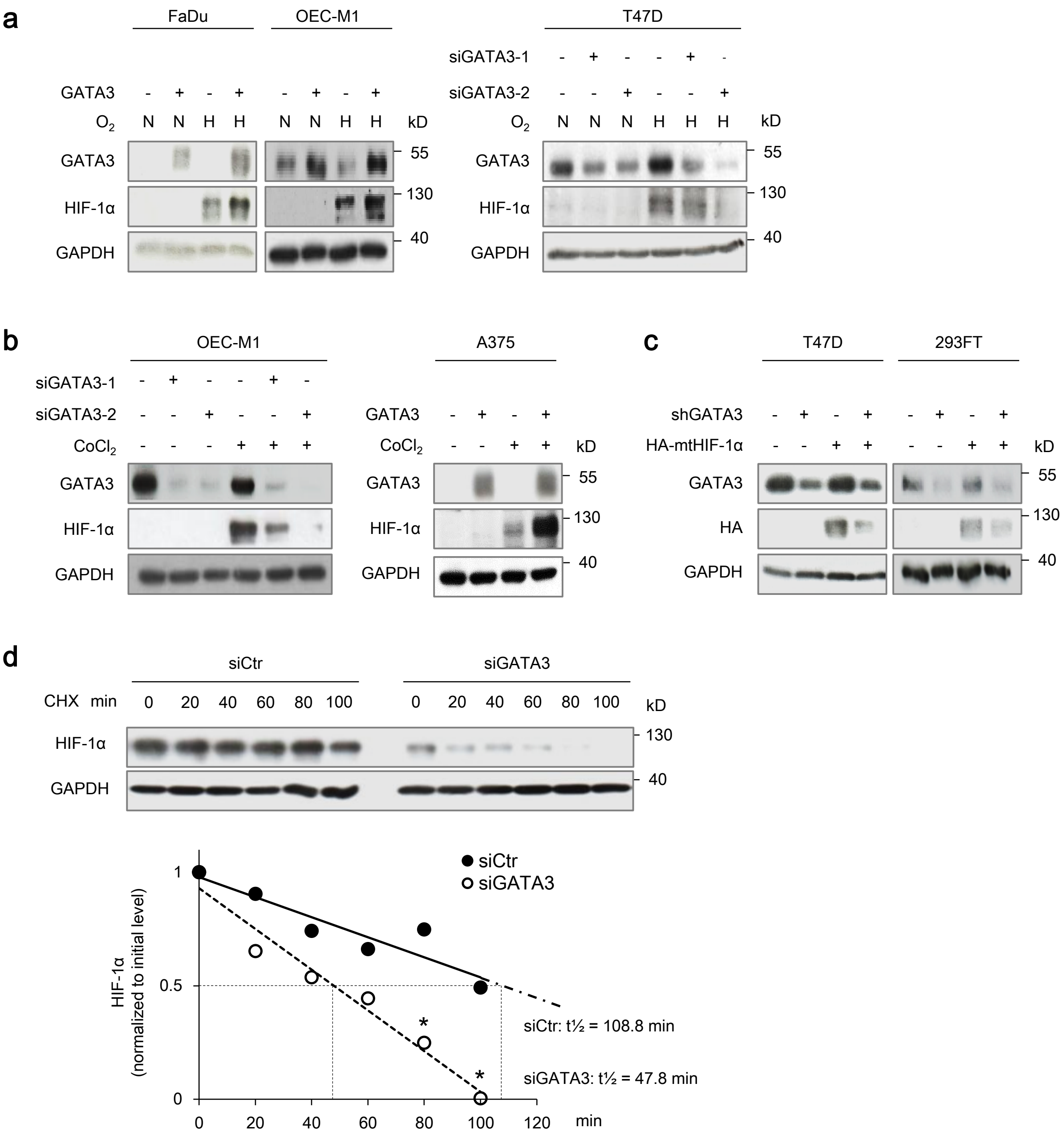

Supplementary Figure S3

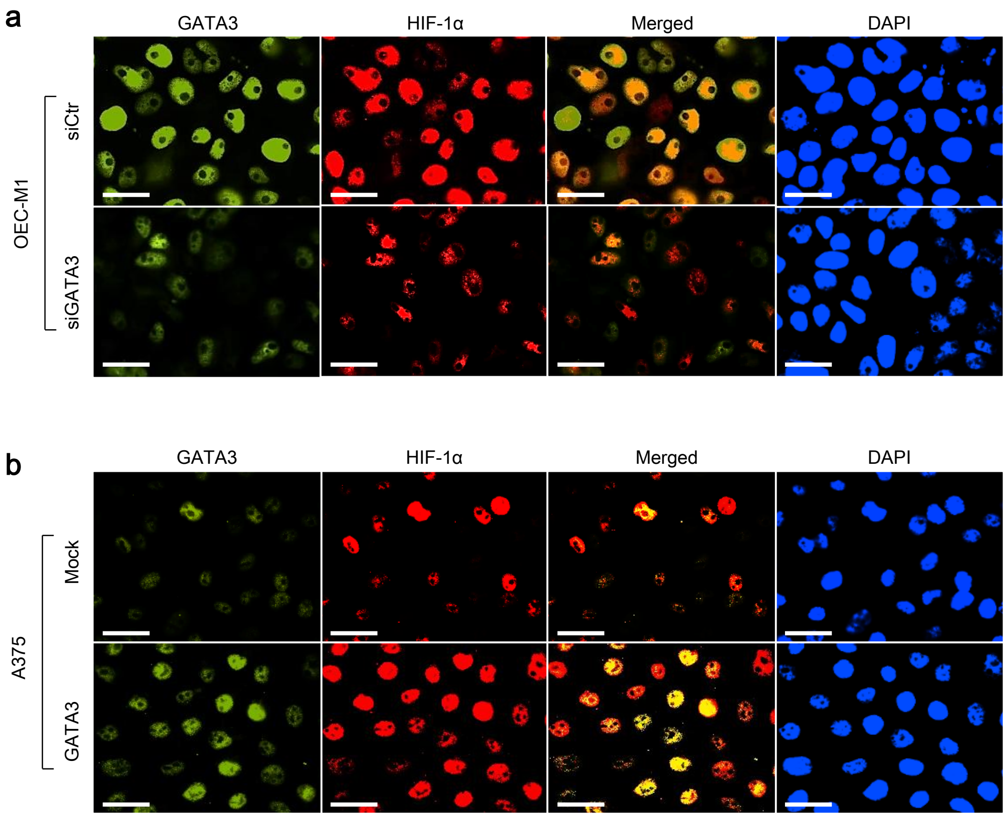

Supplementary Figure S4

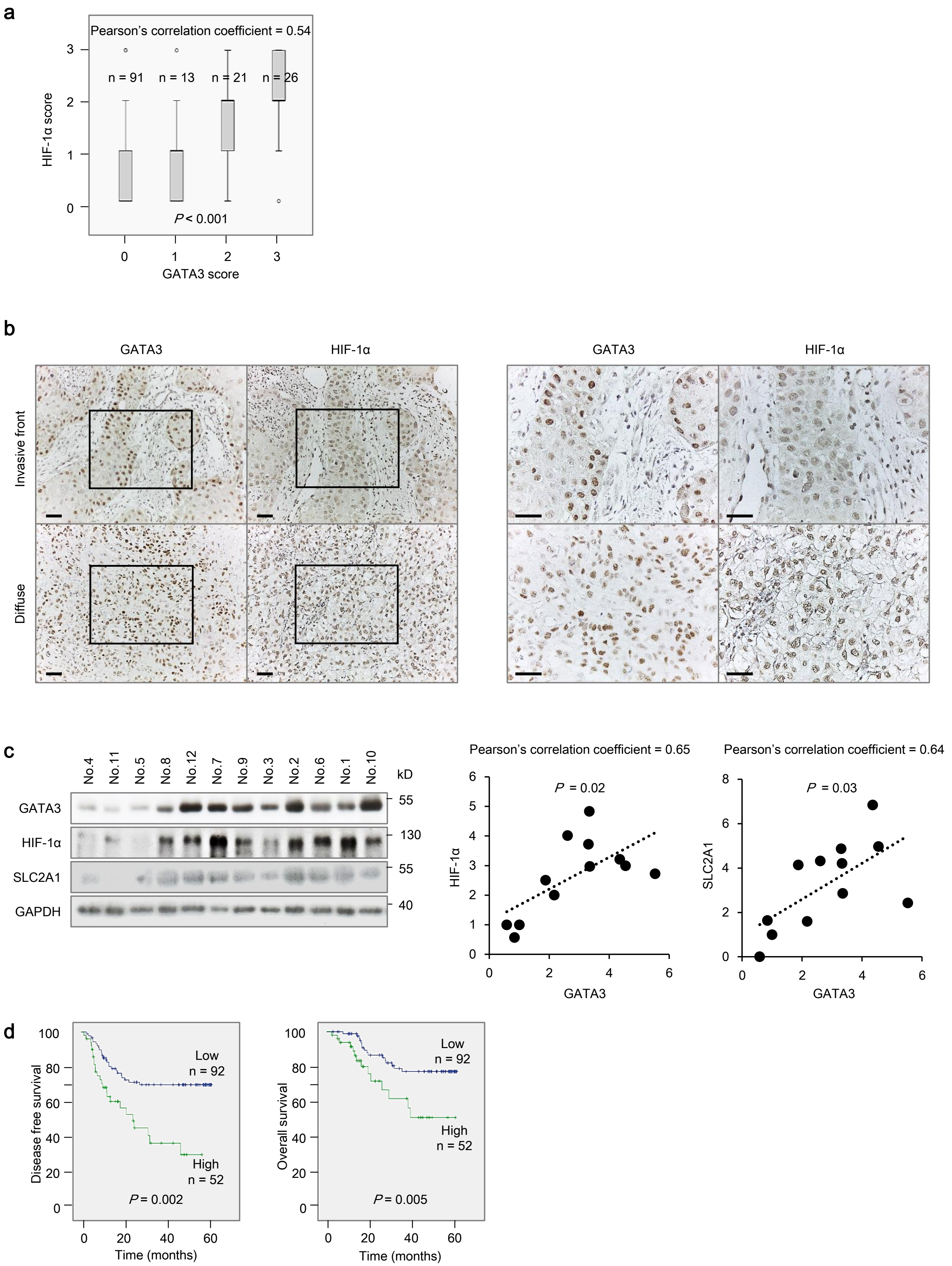

Supplementary Figure S5

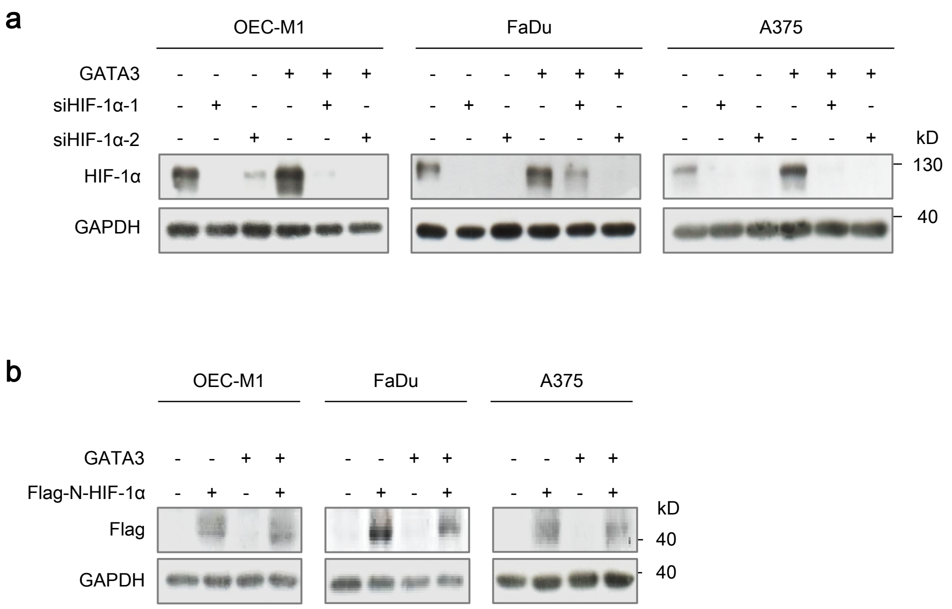

Supplementary Figure S6

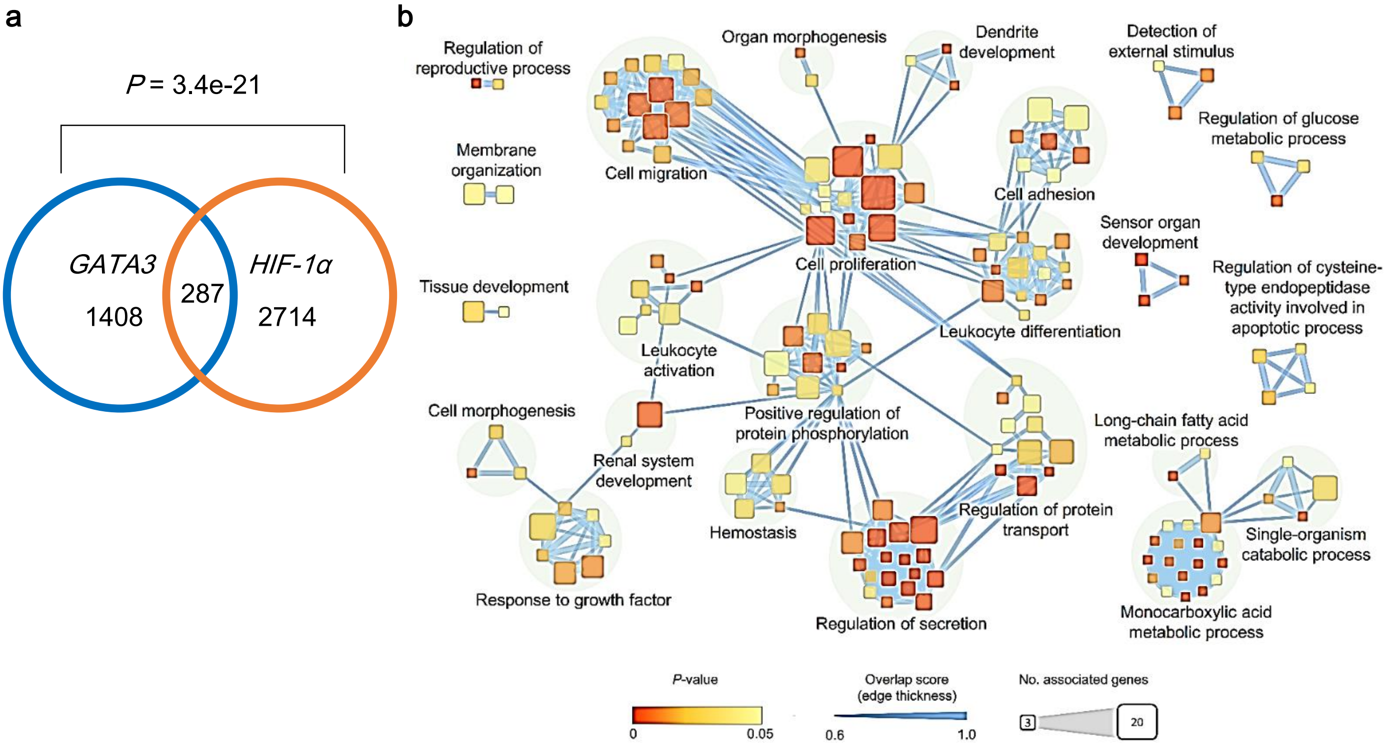

Supplementary Figure S7

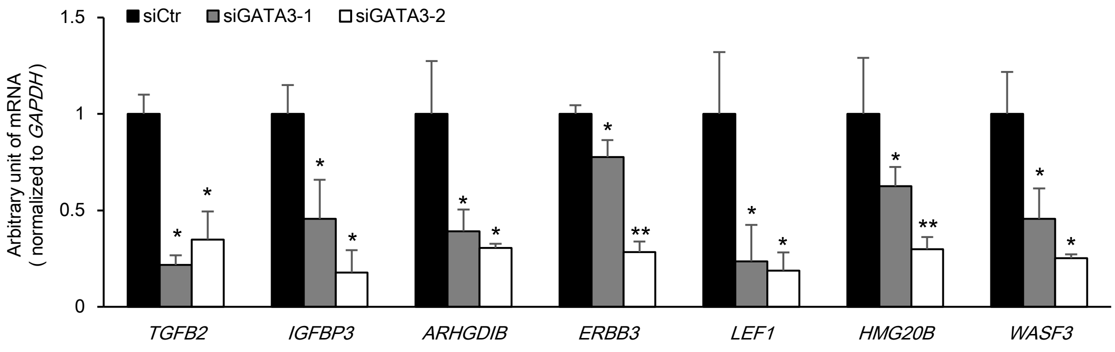

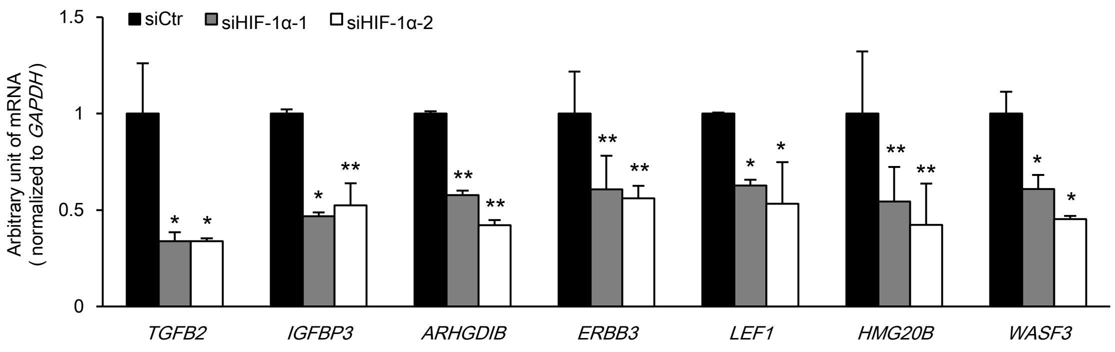

Supplementary Figure S8

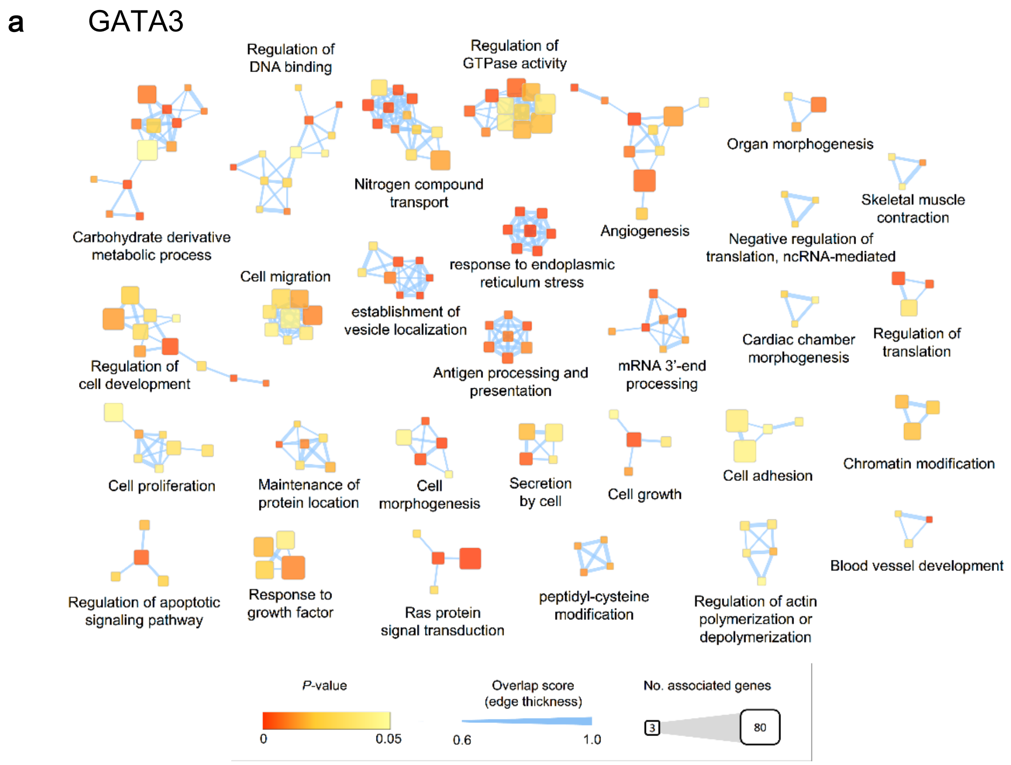

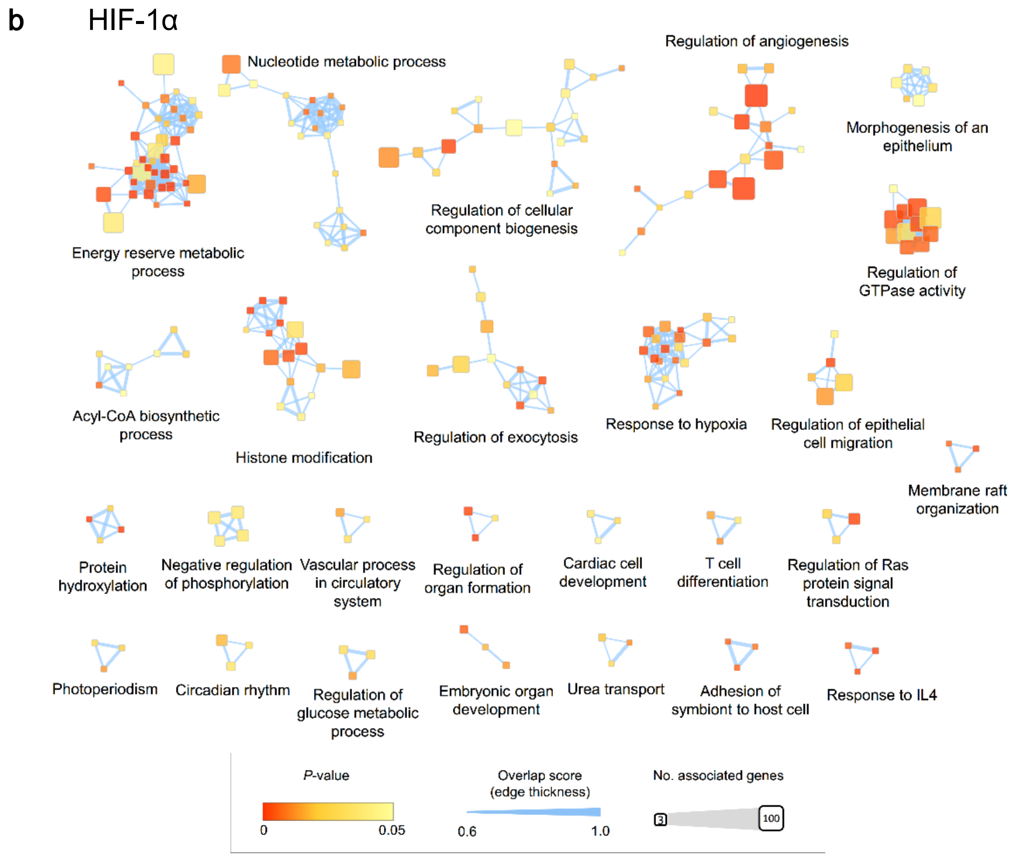

Supplementary Figure S9
