# Supplementary material for: GATA3 interacts with and stabilizes HIF-1α to enhance cancer cell invasiveness
Source: Oncogene. 2017 Mar 6;36(30):4243–52. doi: 10.1038/onc.2017.8 (PMC5537608; doi:10.1038/onc.2017.8)
Supplement: Supplementary Table S1 [file onc20178x2.pdf]

Supplementary Table S1. Cox regression analyses of GATA3 with disease-free survival and overall survival

Analysis	Disease-free survival				Overall survival			
	Univariate		Multivariate		Univariate		Multivariate	
	HR (95% CI)	*P* value	HR (95% CI)	*P* value	HR (95% CI)	P value	HR (95% CI)	*P* value
Age	0.72 (0.40-1.29)	0.27	NT		0.74 (0.39-1.41)	0.35	NT	
Gender	1.56 (0.56-4.33)	0.39	NT		1.66 (0.51-5.37)	0.40	NT	
T	1.94 (1.12-3.36)	0.02*	1.18 (0.66-2.13)	0.57	2.13 (1.15-3.93)	0.02*	1.35 (0.69-2.63)	0.38
N	1.95 (1.12-3.39)	0.02*	0.71 (0.35-1.42)	0.33	2.65 (1.46-4.82)	< 0.001*	1.09 (0.50-2.37)	0.83
M	7.01 (3.96-12.43)	< 0.001*	4.49 (2.36-8.55)	< 0.001*	7.54 (4.08-13.94)	< 0.001*	4.92 (2.45-9.88)	< 0.001*
Grade	1.64 (0.94-2.85)	0.08	NT		1.39 (0.76-2.54)	0.28	NT	
LVI	2.59 (1.50-4.48)	< 0.001*	1.35 (0.62-2.94)	0.45	3.06 (1.68-5.55)	< 0.001*	1.15 (0.47-2.79)	0.76
PNI	3.17 (1.83-5.49)	< 0.001*	2.01 (1.07-3.77)	0.03*	2.79 (1.53-5.09)	< 0.001*	1.80 (0.93-3.49)	0.08
ECS	2.74 (1.57-4.80)	0.001*	1.22 (0.54-2.78)	0.63	3.62 (1.98-6.61)	< 0.001*	1.49 (0.56-3.97)	0.43
GATA3	3.15 (1.84-5.38)	0.001*	2.04 (1.14-3.66)	0.02*	2.09 (1.14-3.82)	0.02*	1.23 (0.65-2.32)	0.53

CI, confidence interval; LVI, lymphovascular invasion; PNI, perineural invasion; ECS, extracapsular spread; NT, not tested. * $P < 0.05$.
