# Supplementary material for: GATA3 interacts with and stabilizes HIF-1α to enhance cancer cell invasiveness
Source: Oncogene. 2017 Mar 6;36(30):4243–52. doi: 10.1038/onc.2017.8 (PMC5537608; doi:10.1038/onc.2017.8)
Supplement: Supplementary Table S2 [file onc20178x3.pdf]

Supplementary Table S2. Correlation of GATA3 and HIF-1α distribution in HNSCC tumors

		GATA3 distribution		Total	*P* value
		Invasive front	Diffuse		
HIF-1α distribution	Invasive front	13	1	14	< 0.001
	Diffuse	7	28	35	
Total		20	29	49	
