# Supplementary material for: GATA3 interacts with and stabilizes HIF-1α to enhance cancer cell invasiveness
Source: Oncogene. 2017 Mar 6;36(30):4243–52. doi: 10.1038/onc.2017.8 (PMC5537608; doi:10.1038/onc.2017.8)
Supplement: Supplementary Table S3 and Materials and Method [file onc20178x4.docx]

**Supplementary Table S3. Primer sequences**

| Primers for real-time RT-PCR | |
| --- | --- |
| *GATA3* | S: 5’-GTCCTGTGCGAACTGTCAGA-3’ |
|  | AS:5’-CGAGCTGTTCTTGGGGAAGT-3’ |
| *HIF-1α* | S: 5’-ACCTATGACCTGCTTGGTGC-3’ |
|  | AS:5’-GGCTGTGTCGACTGAGGAAA-3’ |
| *PGK1* | S: 5’-TGGACAATGGAGCCAAGTCG-3’ |
|  | AS:5’-CTCCACTTCTGGGCCTACAC-3’ |
| *CA9* | S: 5’-CAGTTGCTGTCTCGCTTGGA-3’ |
|  | AS: 5’CAGCTGTAGCCGAGAGTCAC-3’ |
| *SLC2A1* | S: 5’-TTGGCTCCGGTATCGTCAAC-3’ |
|  | AS: 5’-ACCACACAGTTGCTCCACAT-3’ |
| *SLC2A3* | S: 5’-GGTGGCTGCTTTATGGGACT-3’ |
|  | AS: 5’-GTAAAACCCAGTAGCAGCGG-3’ |
| *VEGFA* | S: 5’-CTGTCTAATGCCCTGGAGCC-3’ |
|  | AS: 5’-ACGCGAGTCTGTGTTTTTGC-3’ |
| *TGFB2* | S: 5’-CTACCTGCAGCACACTCGAT-3’ |
|  | AS: 5’-TGGCATCAAGGTACCCACAG-3’ |
| *IGFBP3* | S: 5’-CGCCAGCTCCAGGTGAG-3’ |
|  | AS: 5’-GGGGTGGAACTTGGGATCAG-3’ |
| *ARHGDIB* | S: 5’-CCCTGAAAGAGCTGCAGGAA-3’ |
|  | AS: 5’-GGCTTTCGGATCTGTCACCA-3’ |
| *ERBB3* | S: 5’-GAGGGACCCAGGTCTACGAT-3’ |
|  | AS: 5’-TCACGATGTCCCTCCAGTCA-3’ |
| *LEF1* | S: 5’-GGATTCAGGCAACCCTACCC-3’ |
|  | AS: 5’-TAGGCAGCTGTCATTCTTGGA-3’ |
| *HMB20B* | S: 5’-AGGAGCCGGTGAAGAAACG-3’ |
|  | AS: 5’-TTGGTGATCTCGGGAAAGGG-3’ |
| *WASF3* | S: 5’-GCGCCAGGAGTGGAATATGA-3’ |
|  | AS: 5’-TTGGGAGTAGCCGGGTAAGA-3’ |
| *GAPDH* | S: 5’-ACAGTCAGCCGCATCTTCTT-3’ |
|  | AS: 5’-GACAAGCTTCCCGTTCTCAG-3’ |
| Primers for ChIP assays | |
| *SLC2A1* ([1](#_ENREF_1)) | S: 5’-GAGGCAGAGAACTGCTTGAATCCG-3’ |
|  | AS: 5’-GAACCAGCTCAACTGTATACTGGC-3 |
| *VEGFA* ([2](#_ENREF_2)) | S: 5’-CAGGAACAAGGGCCTCTGTCT-3’ |
|  | AS: 5’-TGTCCCTCTGACAATGTGCCATC-3’ |
| Primers for cloning | |
| *F-HIF-1α* | S: 5’-GGACCGATTCACCATGGAGG-3’ |
|  | AS: 5’-GGATCCGTTAACTTGATCCAAAGCTC-3’ |
| *N-HIF-1α* | S: 5’-GGACCGATTCACCATGGAGG-3’ |
|  | AS: GGATCCGGCCAGCAAAGTTAAAGCAT-3’ |
| *C-HIF-1α* | S: 5’-ATGCCAGCCGCTGGAGACACAAT-3’ |
|  | AS: 5’-GGATCCGTTAACTTGATCCAAAGCTC-3’ |

**Supplementary Figure Legends**

**Supplementary Figure S1.** Effects of GATA3 on cell viability, tumor metastasis, and tumor microvessel densities. (**a**) MTT assays of OEC-M1, FaDu, and A375 cells under normoxia (N) or hypoxia (H). OEC-M1 cells were transfected with non-targeting siRNA (siCtr) or siRNA against *GATA3* (siGATA3-1 or siGATA3-2). FaDu and A375 cells were transfected with empty vector (Mock) or *GATA3*/pcDNA3.1 (GATA3) plasmid. Cells were seeded after transfection for 48 hours. The results are represented as mean ± SD. (**b**) Establishment of GATA3 knockdown and GATA3 overexpressing cells. GATA3 was stably overexpressed in OEC-M1 and A375 cells and stably knocked down in OEC-M1 cells, confirmed by Western blotting. GAPDH was an internal control. (**c**) Effects of GATA3 on tumor metastasis *in vivo*. Upper, representative images of lungs and metastatic tumor nodules indicated by arrowheads. Lower, statistical analysis of tumor nodules. Mock or shGATA3 expressing OEC-M1 stable transfectants (2 × 10^6^ cells / per injection) were injected through tail veins into NOD-SCID mice (n = 7). Results are represented as mean ± SD. Data are analyzed by Student *t*-test. ***P* < 0.01. (**d**) Effects of GATA3 on tumor growth *in vivo*. Upper, images of subcutaneous xenografts. Scale bar, 1 cm. Lower, tumor growth curves. Mock or GATA3 overexpressing OEC-M1 stable transfectants (5 × 10^6^ cells / per injection) were subcutaneously injected into either thigh of the same NOD-SCID mouse (n = 5). Tumor sizes were measured every three days. Results are represented as mean ± SD. Data are analyzed by paired Student *t*-test. **P* < 0.05. (**e**) HIF-1α staining scores in Mock and GATA3 overexpressing OEC-M1 tumor xenografts. The results are represented as mean ± SD from all xenografts. ***P* < 0.01. (**f**) Microvessel density in Mock and GATA3 overexpressing OEC-M1 tumor xenografts. Density was estimated by the number of CD31 positive microvessels per field. The results are represented as mean ± SD from all xenografts. ***P* < 0.01. Scale bars, 50 µm.

**Supplementary Figure S2.** Correlation of GATA3 with tumor cell proliferation and effect of GATA3 on cell viability. (**a**) Representative images of immunohistochemistry result of Ki-67 staining. Ki-67 scores are based on percentage of Ki-67 positive cells within a tumor. 0 = 0%, 1 = 1~33%, 2 = 34~66, 3 = 67~100%. (**b**) Correlation of GATA3 (horizontal axis) and Ki-67 (vertical axis) in HNSCC tumors (n = 56). Pearson’s correlation analysis showed a positive correlation between GATA3 and HIF-1α expression. (**c**) MTT assays. OEC-M1 cells were transfected with non-targeting siRNA (siCtr) or siRNA against *GATA3* (siGATA3-1 or siGATA3-2). FaDu and A375 cells were transfected with empty vector (Mock) or *GATA3*/pcDNA3.1 (GATA3) plasmid. Cells were seeded after transfection for 48 hours. The results are represented as mean ± SD. * *P* < 0.05.

**Supplementary Figure S3.** GATA3 increases HIF-1α protein levels by delaying degradations. (**a**) Western blot analysis of GATA3 and HIF-1α under normoxia (N) or hypoxia (H) for overnight. GAPDH was an internal control. FaDu and OEC-M1 cells were transfected with Mock or GATA3 plasmid. T47D cells were transfected with non-targeting siRNA (siCtr) or siRNA against *GATA3* (siGATA3-1 or siGATA3-2). (**b**) Western blot analysis of GATA3 and HIF-1α in cells treated with or without CoCl_2_ (800 μM) for 4 hours, as indicated. GAPDH was an internal control. OEC-M1 cells were transfected with non-targeting siRNA (siCtr) or siRNA against *GATA3* (siGATA3-1 or siGATA3-2). A375 cells were transfected with Mock or GATA3 plasmid. (**c**) Western blot analysis of GATA3 and P402A/P564A HIF-1α mutant. GAPDH was an internal control. pLKO or sh*GATA3*/pLKO (shGATA3) expressing T47D and 293FT stable transfectants were transfected with empty vector or HA-P402A/P564A *HIF-1α*/pcDNA3.1 (HA-mtHIF-1α). (**d**) HIF-1α degradation assays in OEC-M1 cells. Western blot analysis of HIF-1α in cells transfected with non-targeting siRNA (siCtr) or siRNA against *GATA3* (siGATA3-2). Cells were incubated with CoCl_2_ (800 μM) for 4 hours and then treated with a protein synthesis inhibitor, cycloheximide (CHX, 100 μM). Cell lysates were collected from 0 to 100 minutes after CHX treatment in a 20-minute interval. Upper panel, Western blots of HIF-1α. GAPDH was an internal control. Lower panel, HIF-1α proteins were quantified and half-lives were estimated. The results are represented as mean from three independent experiments. **P* < 0.05.

**Supplementary Figure S4.** Correlation of GATA3 with HIF-1α expression in cells. (**a, b**) Immunofluorescence confocal microscopy of GATA3 (green) and HIF-1α (red) expression in GATA3 knockdown OEC-M1 cells and GATA3 overexpressing A375 cells. Nuclei were stained by DAPI (blue). Colocalization of GATA3 and HIF-1α was shown in merged images. The negative control did not show specific signals (data not shown). Scale bars, 50 μm.

**Supplementary Figure S5.** Correlation of GATA3 with HIF-1α expression in clinical samples. (**a**) Correlation of GATA3 (horizontal axis) and HIF-1α (vertical axis) in HNSCC tumors (n = 151). Pearson’s correlation analysis showed a positive correlation between GATA3 and HIF-1α expression. (**b**) Representative images of distribution of GATA3 and HIF-1α. Right panel represents enlarged images from black boxes in left panel. Scale bars, 50 μm. (**c**) Correlation of GATA3, HIF-1α, and SLC2A1 expression in primary HNSCC tumor tissues (n = 12). Left panel, Western blots of GATA3, HIF-1α, and SLC2A1. GAPDH was an internal control. Middle and right panels, Pearson’s correlation analysis between GATA3 (horizontal axis) and HIF-1α (vertical axis) or between GATA3 (horizontal axis) and SCL2A1 (vertical axis). (**d**) Kaplan-Meier survival analysis. Patients with follow-up period over 18 months are included (n = 144). For assessment of HIF-1α intensity, each field was graded semi-quantitatively on tree-tier scale where 0 = none staining, 1 = weak staining, 2 = moderate staining, 3 = strong staining. Left and right panels indicate disease-free and overall survivals of patients with low (score = 0, 1) and high (score = 2, 3) HIF-1α expression, respectively.

**Supplementary Figure S6.** Western blot analysis of HIF-1α and N-HIF-1α expression in Mock or GATA3 overexpressing OEC-M1, FaDu, and A375 stable transfectants. (**a**) Mock or GATA3 overexpressing OEC-M1, FaDu, and A375 stable transfectants were transfected with non-targeting siRNA or siRNAs against *HIF-1α* (siHIF-1α-1 and siHIF-1α-2). Cells were incubated with CoCl_2_ (800 μM) for 4 hours. (**b**) Mock or GATA3 overexpressing OEC-M1, FaDu, and A375 stable transfectants were transfected with empty vector or *N-HIF-1α*/pCMV-Tag 4A (Flag-N-HIF-1α). Cells were incubated with CoCl_2_ (800 μM) for 4 hours. GAPDH was an internal control.

**Supplementary Figure S7.** GATA3 and HIF-1α co-regulated genes under hypoxia. (**a**) Venn diagram depicting numbers of differentially expressed genes in *GATA3*- and *HIF-1α*-knockdown OEC-M1 cells, 1408 and 2714, respectively. The *P* value was obtained from Fisher’s exact test. siGATA3-2 and siHIF-1α-2 were used for GATA3 and HIF-1α knockdown, respectively. (**b**) Functional map of the common set of *GATA3/HIF-1α*-regulated genes (287 genes). A node denotes the enriched GO term (*P* < 0.05) and an edge represents the gene overlap score between nodes > 0.6. Node color encodes the enriched *P* value and node size is proportional to the number of genes which are associated with a given GO term. Edge thickness is proportional to the overlap score. Groups of functionally related GO terms are manually identified and labelled with the appropriate terms.

**Supplementary Figure S8.** Real-time RT-PCR validations for GATA3/HIF-1α regulated genes in microarray analysis. OEC-M1 cells were transfected with non-targeting siRNA, *GATA3* siRNAs (siGATA3-1, siGATA3-2), or *HIF-1α* siRNAs (siHIF-1α-1, siHIF-1α-2), as indicated. Real-time RT-PCR analysis of the expression of genes, including *TGFB2, IGFBP3, ARHGDIB, ERBB3, LEF1, HMG20B*, and *WASF3*.

**Supplementary Figure S9.** Functional maps of GATA3 and HIF-1α regulated genes. (**a**) Functional maps of GATA3 regulated genes in OEC-M1 cells under hypoxia. (**b**) Functional maps of HIF-1α regulated genes in OEC-M1 cells under hypoxia.

**Supplementary Materials and Methods**

**MTT assay**

Cells (2×10^3^) were seeded in 96-well plates. After culturing for a specific time period, 3-(4,5 dimethyl-2 thiazolyl)-2,5 diphenyl-2H tetrazolium bromide solution (MTT, .5 mg/ml, Sigma[-Aldrich](http://www.sigmaaldrich.com/catalog/product/sigma/m2128)) was added. After 3 hours of incubation at 37°C, the formazan crystals were dissolved by adding 10% sodium dodecyl sulfate (SDS) containing 0.01 M HCl. The absorbance was measured at the dual wavelengths of 550 and 630 nm with a spectrophotometer.

**Protein degradation assay**

OEC-M1 cells were incubated with CoCl_2_ (800 μM) for 4 hours before cycloheximide (CHX, 100 μM) were added. Lysates were harvested every 20 minutes from 0-100 minutes after addition of cycloheximide and subjected for Western blot analysis. Protein bands were quantified by ImageJ software.

**Immunofluorescence confocal microscopy**

A375 and OEC-M1 cells were seeded on chamber slides and subjected to CoCl_2_ treatment for 4 hours. The cells were fixed with 4% paraformaldehyde and cell membranes were permeated with 1% Triton X-100. For blocking, 5% BSA/PBS was used. Primary antibodies against GATA3 (Santa Cruz Biotechnology) and HIF-1α ([Novus Biologicals](http://www.novusbio.com/HIF-1-alpha-Antibody-H1alpha67_NB100-105.html)) were added and incubated at 4°C for 18 hours. After wash, Cy3-conjugated goat anti-rabbit IgG and FITC-conjugated goat anti-mouse IgG (Jackson Immunoresearch) were added and incubated for 1 hour at room temperature. The nuclei were counterstained by 4’,6-diamidino-2-phenylindole (DAPI).

**Differential expression analysis and functional analysis**

Total RNA was extracted using GeneJET RNA Purification kit (Thermo Fisher Scientific) following the manufacturer's protocol and quantified by NanoDrop spectrophotometer (Bio-Rad). The RNA quality was monitored with Agilent 2100 Bioanalyzer (Agilent Technologies, Santa Clara, CA). cDNA prepared from 10 μg of total RNA was labeled with aa-dUTP using Invitrogen SuperScriptTM Plus Indirect cDNA Labeling System according to the manufacturer's protocol, followed by aa-cDNA column purification (QIAGEN, Valencia, CA). Alexa/CyDye was incorporated to aa-cDNA followed by column purification with Alexa/CyDye-cDNA cRNA purification (Qiagen). DNA yields were confirmed by 1% DNA agarose gel and visualized with Fuji image reader at 600V PMT. Agilent Gene Expression Hybridization Kits was used for hybridization according to the manufacturer’s instruction. Briefly, 16 μl of dye labeled cDNA in water was fragmented at 98°C for 3 mins in a reaction volume of 40 μl containing 4 μl Agilent 10× blocking agent and 20 μl of Agilent 2×GExHybridization Buffer HI-RPM and hybridized to Agilent SurePrint G3 Human Gene Expression 8×60K v2 Microarray (G4851B) at 65°C and rotated at 10 rpm for 17 hours. After hybridization, microarrays were washed 1 minute at room temperature with GE Wash Buffer 1 (Agilent) and 1 minute with 37°C GE Wash buffer 2 (Agilent), then dried immediately by brief centrifugation. The microarrays were scanned on the Agilent DNA Microarray Scanner (US9230696) using one color scan setting for 8×60k array slides. The scanned images were analyzed with Feature Extraction Software 10.5.1.1 (Agilent). Features flagged in Feature Extraction as Feature Non-uniform outliers were excluded. Background correction, quantile normalization, and differential expression analysis of microarray datasets were performed using the limma R package ([4](#_ENREF_4)). The genes with *P* < 0.05 were considered as differentially expressed genes. The Gene Ontology (GO) enrichment analysis was applied to the differentially expressed genes. The Fisher’s exact test was used to assess whether a GO term was enriched in differentially expressed genes. The enriched GO terms (*P* < 0.05) were graphically organized into a network, where a node denotes each GO term and an edge represents gene overlap between GO terms([5](#_ENREF_5)). The gene set overlap was scored by the arithmetic average of Jaccard coefficient JC = $\frac{|A\cap B|}{|A\cup B|}$ and Simpson coefficient SC = $\frac{|A\cap B|}{\min(|A|,|B|)}$ in which *A* and *B* are two gene sets. An edge which overlap score passes a threshold was presented in the networks. The networks were visualized by Cytoscape ([6](#_ENREF_6)).

**Supplementary References**

1. Ohtsubo K, Chen MZ, Olefsky JM, Marth JD. Pathway to diabetes through attenuation of pancreatic beta cell glycosylation and glucose transport. Nat Med 2011;17(9):1067-75.

2. Cascio S, D'Andrea A, Ferla R, Surmacz E, Gulotta E, Amodeo V, et al. miR-20b modulates VEGF expression by targeting HIF-1 alpha and STAT3 in MCF-7 breast cancer cells. J Cell Physiol 2010;224(1):242-9.

3. Yan Q, Bartz S, Mao M, Li L, Kaelin WG, Jr. The hypoxia-inducible factor 2alpha N-terminal and C-terminal transactivation domains cooperate to promote renal tumorigenesis in vivo. Mol Cell Biol 2007;27(6):2092-102.

4. Ritchie ME, Phipson B, Wu D, Hu Y, Law CW, Shi W, et al. limma powers differential expression analyses for RNA-sequencing and microarray studies. Nucleic Acids Res 2015;43(7):e47.

5. Merico D, Isserlin R, Stueker O, Emili A, Bader GD. Enrichment map: a network-based method for gene-set enrichment visualization and interpretation. PLoS One 2010;5(11):e13984.

6. Shannon P, Markiel A, Ozier O, Baliga NS, Wang JT, Ramage D, et al. Cytoscape: a software environment for integrated models of biomolecular interaction networks. Genome Res 2003;13(11):2498-504.
